# Potential of Nitrogen Gas (N_2_) Flushing to Extend the Shelf Life of Cold Stored Pasteurised Milk

**DOI:** 10.3390/ijms14035668

**Published:** 2013-03-11

**Authors:** Patricia Munsch-Alatossava, Abdul Ghafar, Tapani Alatossava

**Affiliations:** Department of Food and Environmental Sciences, P.O. Box 66, FIN-00014 University of Helsinki, Finland; E-Mails: abdul.ghafar@helsinki.fi (A.G.); tapani.alatossava@helsinki.fi (T.A.)

**Keywords:** pasteurised milk, cold storage, shelf life, modified atmosphere, N_2_ gas, *Bacillus* spp., spores

## Abstract

For different reasons, the amount of food loss for developing and developed countries is approximately equivalent. Altogether, these losses represent approximately 1/3 of the global food production. Significant amounts of pasteurised milk are lost due to bad smell and unpleasant taste. Currently, even under the best cold chain conditions, psychrotolerant spore-forming bacteria, some of which also harbour virulent factors, limit the shelf life of pasteurised milk. N_2_ gas-based flushing has recently been of interest for improving the quality of raw milk. Here, we evaluated the possibility of addressing bacterial growth in pasteurised milk during cold storage at 6 °C and 8 °C. Clearly, the treatments hindered bacterial growth, in a laboratory setting, when N_2_-treated milk were compared to the corresponding controls, which suggests that N_2_-flushing treatment constitutes a promising option to extend the shelf life of pasteurised milk.

## 1. Introduction

The world today confronts simultaneously several challenges which more than ever require new strategies to develop sustainable food systems. In a world that faces on-going climate change, the risk of finite resources, and where the current state of food-borne diseases may be summarised in the following way “the challenges of 20 years ago persist while new ones continue to emerge” [1], all steps of food production, storage, and processing through distribution are being challenged.

The issue of whether the world’s current food production is sufficient is debatable. Some argue that the continuing population and consumption increase implies that the global food demand will need to increase by 70% to 100% [2]. Others believe that global food production only suffers from serious deviations [3], but all parties have highlighted the tremendous waste from production to consumption for developing and developed countries. Roughly one-third of the edible parts of food produced for human consumption is lost or wasted [4]. The wastage of milk at the consumption level is very high in industrialised countries. For British households, it is estimated that 38% of milk is discarded because of bad smell/taste and 37% because it has expired [5]. In developing regions, wastage of milk occurs mainly during postharvest handling, storage, and distribution [4].

According to the FAO, approximately 80% of the milk consumed worldwide is obtained outside of the set standards; cold chain-based storage is generally not applicable or affordable. In developed countries, on the other hand, an effective cold chain that ensures food safety from production until processing has spoiling bacteria which cause losses to the dairy industry. Psychrotrophic bacteria, which are capable of growing below 7 °C and which are present in raw milk, belong to numerous genera. These bacteria are known for producing extra-cellular enzymes (proteases, lipases, phospholipases) many of which can withstand the classical heat treatments of milk [6]. With some exceptions (such as *B. cereus* or *Listeria* strains), most of these bacteria are considered benign. However, recent observations have shown that raw milk bacteria, whether mesophiles or psychrotrophs from conventional or organic production systems, carry antibiotic resistance (AR) features at levels that raise the question of whether cold storage of milk is actually promoting AR amplification [7–9].

In general, unpasteurised milk is considered a public health threat. Heat treatments, such as pasteurisation, aim to preserve food materials from spoilage and disease-creating microorganisms. Pasteurisation of raw milk can be achieved by several combinations of time and temperature, with the most widely applied comprising a 15 second heat treatment at 72 °C (HTST: high temperature, short time method; [10]). The treatments target two important pathogenic non-spore forming bacterial species present in raw milk (*Mycobacterium tuberculosis* and *Coxiella burnetti*) among the most heat resistant, and they destroy yeasts and moulds, Gram-negative bacteria as well as many Gram-positive bacteria. Pasteurisation seems however not to be the ultimate tool to control milkborne pathogens considering the increasing number of incidences in which foodborne pathogens are detected in fluid milk and ready-to-eat dairy products [11]. Although the standard for the bacteriological limit (expected to be below 100,000 cfu/mL prior to commingling with milk from another producer and not over 300,000 cfu/mL prior to pasteurisation, [12,13]) for raw milk in developed countries appears to be consensual, the limit for bacterial counts in pasteurised milk is expected to be below 20,000 cfu/mL in the United States (“Grade A” pasteurised milk) and should be below the range 5000 and 50,000 cfu/mL in Europe [12,13].

The shelf life of HTST pasteurised milk is estimated to range from 7 to 28 days [10]. In the US, the shelf life of fluid milk is between 1 to 3 weeks [14], whereas in Brazil, for example, the range drops to 3–8 days, mainly due to poor cold chain conditions [15].

If Gram-negative bacteria are encountered in pasteurised milk, their presence is usually indicative of failed pasteurisation or post-pasteurisation contamination; nevertheless, under the best pasteurisation conditions, bacterial growth and the resulting spoilage constitutes the major limiting factor in extending the shelf life of conventional HTST pasteurised milk [6,16,17]. Gram-positive psychrotolerant bacterial species are mainly implicated; different genera enter the raw milk as spores, survive the pasteurisation steps and germinate. Due to the ability of these bacteria to grow while producing spoilage enzymes at refrigeration temperatures, the degradation of milk components causes off-flavours or curdling of the milk. Extensive studies in the US have shown that, for cold stored HTST pasteurised milk, *Bacillus* spp. dominates in the early stages of cold storage (<7 days), whereas *Paenibacillus* spp. becomes predominant in the later stages (>10 days) [16–18].

The application of modified/controlled atmospheres to improve food storage is ancient; one early application of elevated CO_2_ involved the preservation of meat carcasses on their way from New Zealand to Great Britain in the 1930s [19]. Numerous studies have reported the inhibition of bacterial growth following the addition of carbon dioxide to raw milk [20–24]. However, the treatments also promote the acidification of milk or the modification of its sensory properties. Two studies investigated the use of nitrogen (N_2_), considered an inert gas, to inhibit bacterial growth and the spoilage potential present in raw milk [24,25]. Both studies investigated a “closed system” with no possibility of gas exchange between the milk flasks and the environment. Recently, we investigated the possibility of applying N_2_ gas to raw milk that was cold stored in bottles/tanks and kept in an “open system”. We observed that the treatments especially targeted phospholipases producers, including *Bacillus cereus*. Although not yet optimised, N_2_ gas flushed into raw milk constitutes an interesting perspective to improve raw milk quality at both laboratory and pilot plant scales [26–29].

To further investigate the applicability of N_2_ gas at the level of fluid milk, in the present study, we considered pasteurised milk that was stored and treated similarly to raw milk at laboratory scale and we examined the impact of the N_2_-treatments on bacterial growth.

## 2. Results and Discussion

### 2.1. Microbiological Analyses

#### 2.1.1. Retail Pasteurised Milk

**Figure 1:**
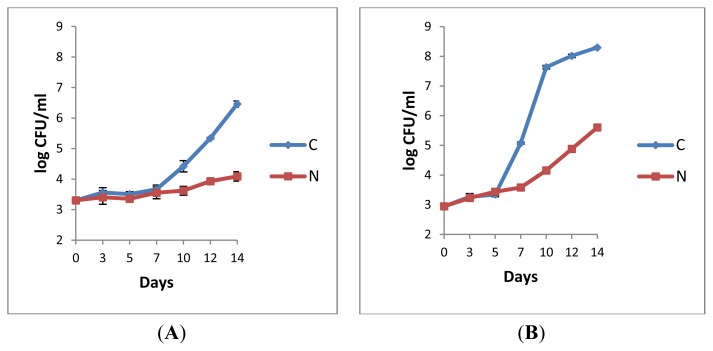
Total aerobic bacterial counts (log cfu/mL) on plate count agar (PCA) from retail pasteurised milk sample P3 stored at 6 °C (**A**), and from sample P6 stored at 8 °C (**B**). (C: control, N: N_2_ flushing). Error bars = ± SD from triplicate platings.

For both control and N_2_-flushed pasteurised milks maintained at 6 °C, the total counts (approximately 3.30 log cfu/mL at day 0, Figure 1A) remained the same until day 7. Then, a gradual increase in bacterial counts was observed for the control (C), contrary to the treated sample (N), for which only a moderate growth (approximately 0.5 log unit) was recorded over 14 days (Figure 1A). For the samples stored at 8 °C, at first, the bacterial growth was low for both conditions (Figure 1B). After 7 days in storage, at every sampling day, the counts from flushed milk (N) were at least 2.9 log units lower than for the control (C) (Figure 1B). Clearly, the N_2_ treatment impacted the rate of bacterial growth.

The fact that any temperature rise, even minor, favours bacterial growth in milk is well known [6]. The comparison of the differences of counts in log-units between initial and final sampling points between C and N (3.2/C and 0.7/N, Figure 1A, and 5.4/C and 2.7/N, Figure 1B) showed that the hindrance of bacterial growth was best realised by combining storage at lower temperatures together with N_2_ flushing. At the level of pasteurised milk, additional studies are required to determine the limitations of N_2_-gas treatment efficiency imposed by the storage temperatures. As already observed for raw milk, N_2_-based treatments proved to still be efficient at 12 °C for up to 48 h [27]. The factor within the initial microflora (bacterial types, total load) and the storage temperature that mainly impacts N_2_-treatment efficiency requires further investigation.

#### 2.1.2. Bacterial Counts Following Heat Treatments of Milk Samples

**Figure 2:**
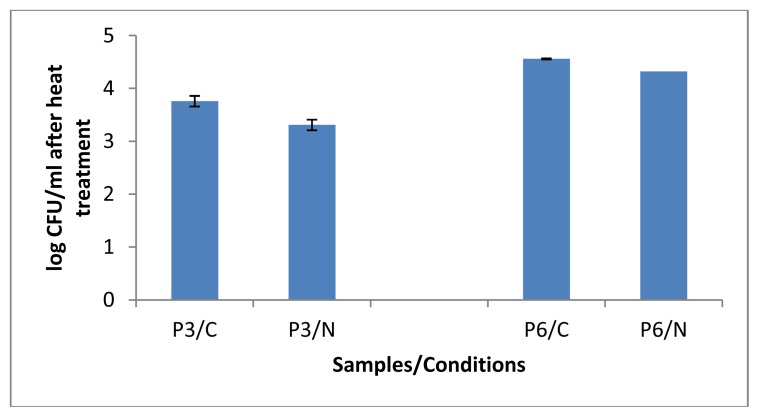
Aerobic bacterial counts (enumerated on BHI (Brain Heart Infusion, Oxoid Ltd., Cambridge, UK) agar) following heat treatment at 75 °C for 15 min for controls (P3/C and P6/C) and N_2_-treated retail pasteurised milks (P3/N and P6/N), withdrawn at the end of the cold storage at 6 °C (P3) and 8 °C (P6). Error bars = ± SD from triplicate platings.

After 14 days of cold storage, both pasteurised milk samples P3 and P6 showed lower counts of heat resistant bacteria (Figure 2) compared to the corresponding total counts (Figure 1A,B). Interestingly, following the heat treatments, the counts retrieved from P3/N and P6/N were slightly lower compared to their corresponding controls (P3/C and P6/N), which suggests that the N_2_ gas flushing treatment applied over 14 days did not stimulate massive sporulation of aerobic bacteria. Considering the considerable diversity of spore-formers present in milk, more studies are needed to investigate whether some species might be sporulating under the N_2_ gas treatment. Nevertheless, preliminary observations from isolates selected from pasteurised milk showed that sporulation did not occur under the N_2_-treatments at 6 °C [30].

#### 2.1.3. Microbiological Analyses of Raw Milk and the Corresponding Pasteurised Milk

**Figure 3:**
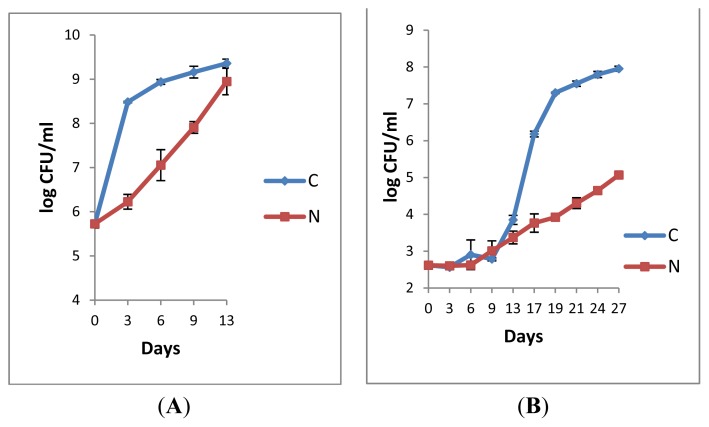

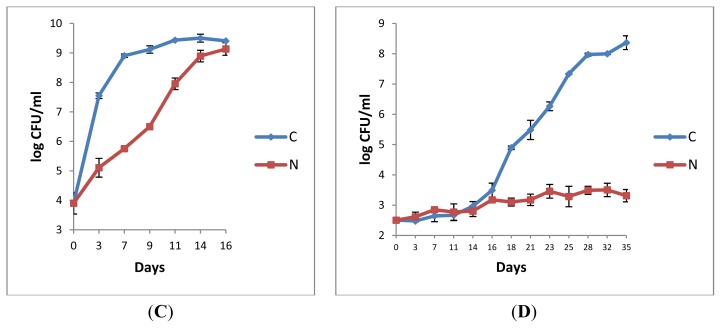
Total plate counts on PCA agar of the paired raw milks L2R (**A**) and L3R (**C**) and the corresponding pasteurised (73 °C/15 s) milks L2P (**B**) and L3P (**D**) during cold storage at 6 °C. (C: control, N: N_2_ flow rate of 120 mL/min). Error bars = ± SD from triplicate platings.

The analyses of the raw milks (L2R and L3R, Figure 3A,C) revealed that bacterial growth was hindered in both samples under N_2_ gas flushing, despite high initial counts for L2R (Figure 3A). Contrary to data from the raw milk analyses [27], a complete inhibitory effect of bacterial growth by N_2_ was not observed here. Nevertheless, the N_2_ treated samples L2R and L3R required a double cold storage time at 6 °C to reach the level of 9 log-units compared to the corresponding controls (Figure 3A,C).

Both samples from the milks pasteurised (73 °C/15 s) at the university pilot plant exhibited rather similar initial bacterial counts (approximately 2.5 log units, Figure 3B,D). No growth was observed, for either condition during the first storage days of L2P; the highest bacterial growth for C was recorded between sampling days 13 and 19 (Figure 3B). Total counts from the N_2_-treated sample L2P gradually increased, but still after 27 days, the counts were approximately 2.9 log-units lower than the control (Figure 3B). For L3P, no growth was observed for both the control and the N_2_-treated milk sample until day 14 of cold storage. Afterwards, the counts continuously increased for the control (Figure 3D). Most interestingly, in this study, the counts enumerated from L3P under continuous N_2_ flushing increased by less than one log-unit only within 35 days cold storage at 6 °C. Until the end of the experiment, the counts remained below the bacteriological limits defined for pasteurised milk.

The comparison of the raw and the corresponding pasteurised milks (Figure 3) suggests that maximum efficiency of the N_2_ treatment is gained at the pasteurised milk stage when the raw milk quality was best *i.e.*, when the rate of bacterial growth was minimal, when the initial bacterial counts were lowest (when the raw milk was most fresh). At the time when the N_2_-flushing treatments started, the retail pasteurised milk samples P3 and P6 had 4 or 5 days of cold storage history (according to the packaging dates), respectively whereas the samples L2P and L3P from the pilot plant were treated shortly after pasteurisation, which could explain a greater delay in bacterial growth in the later cases (irrespective of the conditions, whether C or N) (Figure 3B,D compared to Figure 1A,B). The observation that the efficiency of the N_2_-treatments depended on the “application stage” was already made for raw milks, where the inhibitory effect was maximal when N_2_ gas was applied on fresh raw milks with low initial bacterial counts [27].

The comparison of the N_2_-treatment efficiency at 6 °C and 8 °C (Figure 1A,B and Figure 3B,D) indicates that the inhibitory effects on bacterial growth were higher at the lowest temperature, which is in line with former observations from raw milk [27]. The pH of the pasteurised milk was not affected by the treatments; the initial pH values ranged from 6.69 to 6.87. Following N_2_-flushing, the pH values were between 6.82 and 6.87, compared to 6.60 and 6.87 for the corresponding controls, which indicates that the pH of pasteurised milk is not altered following the continuous application of N_2_, as already found for raw milk [24,27].

### 2.2. Characterisation of Selected Bacterial Isolates by 16 rRNA and rpoB Gene Sequences

The agar plates from control and treated milks harboured greater bacterial diversity at the beginning of cold storage, as less distinct types of bacteria appeared on plates after time had elapsed. A number of bacterial isolates were selected from both control and treated milk samples (Table 1). Colonies that grew under N_2_ gas flushing were preferred in order to identify species that resist the treatment.

The 16S rDNA sequence of P3Nd3, isolated three days after N_2_ flushing, showed 99% sequence similarity with *Microbacterium lacticum* (Table 1). L2PCa21, isolated after three weeks of storage at 6 °C from the control sample L2PC, showed 99% sequence similarity with both 16S rDNA and the *rpoB* gene of *Paenibacillus odorifer* (Table 1). *P. odorifer* was first isolated from wheat roots and pasteurised pureed courgettes [31]; recently, it has been commonly found in pasteurised milk [17].

It has been shown that *Paenibacillus* isolates, which are most prominent at the end of cold storage [16], constitute the key biological barrier together with other *Bacillus* spp., that limits the shelf life of pasteurised milk during cold storage in the US. At the beginning of this study, we anticipated that N_2_ gas flushing would favour the growth of *Paenibacillus* species, as at least eight *Paenibacillus* species (among them *P. odorifer*) harbour nitrogen fixing strains [31,32]. As no *Paenibacillus* was encountered here in N_2_-treated milk, we may hypothesise that *Paenibacillus* bacteria were present in lower amounts in our pasteurised milk samples or were sensitive to N_2_ gas during cold storage. Further studies are required to verify whether N_2_ exerts inhibitory effects against *Paenibacillus* during cold storage.

Based only on the 16S rDNA partial sequence, the taxonomic assignation could not be resolved for the isolates L2PNa, L2PNa13 and L2PNa6, which were attributed to different *Bacillus* species. Due to the high diversity, and hence, complex taxonomy at the *Bacillus* genus level, the identification of the later isolates requests thorough investigations.

Selected from three distinct samples (P3, P6 and L2P), from different sampling times, under conditions C or N, and from different agar plates (to limit “clonality”), the analyses of the partial 16S rDNA sequences revealed that 18 out of 24 isolates showed 99% sequence similarity with the 16S rDNA of *Bacillus weihenstephanensis*, *B. mycoides*, *B. thuringiensis*, and *B. anthracis* (Table 1). Out of one isolate (L2PNb, Table 1), all isolates were selected from milks that were placed in cold storage for more than 7 days (at 6 or 8 °C). All isolates exhibited protease and phospholipase activities; in the tested conditions, also 11 isolates appeared to be lipase producers (Table 1). Comparative analyses of the sequences showed that, despite the intercistronic variability (when considering the 14 *rrn* operons of *Bacillus weihenstephanensis* BcerKBAB4), the majority of the isolates showed total sequence identity with the consensus sequence of the 14 *rrn* operons, and with the 16S rDNA sequence of *B. weihenstephanensis* DSM 11821 (NR_024687.1). The sequence of L2PNb was most divergent, as five nucleotides out of 650 differed from the consensus sequence. The *rpoB* partial gene sequence revealed that 15 of 18 isolates showed 98% to 99% sequence similarity with the *rpoB* gene of *Bacillus weihenstephanensis* (CP000903), further strengthening the assignation to *B. weihenstephanensis* species (Table 1).

*Bacillus weihenstephanensis* was recognised as psychrotolerant and was differentiated from *Bacillus cereus* by its ability to grow at 7 °C but not at 43 °C; the nucleotide sequence ^1003^TCTAGAGATAGA was proposed as a signature of the species [33]. During our analyses, we noticed that this sequence was also present in *Bacillus mycoides* (NR_036880.1). In a recent study, *Bacillus weihenstephanensis* was more often retrieved from raw milk than from pasteurised milk samples [17]. Interestingly, the investigations with the psychrotolerant strain KBAB4 showed that the virulence gene expression was in part determined by temperature; higher virulence and cytotoxicity was observed at 15 °C compared to 30 °C [34].

However, whether *Bacillus weihenstephanensis* is favoured during N_2_-treatment to the detriment of *Paenibacillus* spp. requires further investigations.

Whether N_2_-gas flushing treatments of raw and pasteurised milks enters the category of “green technology” to improve food safety and quality is on first view not questionable, considering that N_2_ gas is an acceptable chemical agent (E941) in organic farming and also in food production systems. The study at a pilot plant scale showed that N_2_ gas, extracted from compressed air, proved to be efficient in improving the quality of raw milk, even though treatments are far from optimised [28]. However, N_2,_ due to its abundance (79% in air), is not a finite resource and can be extracted from the air. To fully meet the sensibility of a “green” technology, the extraction of N_2_ from ambient air must occur in a fully sustainable matter.

The comprehension of the “biocidal effect” requires further investigation. Thus far, at the pasteurised milk level, bacterial growth was “only” inhibited (Figures 1 and 3) as visible from enumerated counts, which reflect the balance of cells that remain viable over dead ones. The major aim here was to investigate whether any inhibitory effect could be detected at all. Due to the use of non-selective agar media, no primary bacterial target was identified in this preliminary study. Conversely, bacterial isolates of *B. weihenstephanensis* survived cold storage periods of 7, 14, and 21 days (Table 1), following N_2_ treatment, and they were encountered in different milk samples.

From studies performed with raw milk in the laboratory and at the pilot plant scale, it appeared that phospholipases (PLases) producers were a clear target of the N_2_ treatment. This group of bacteria appeared to be excluded “sooner or later” by N_2_ treatment at the laboratory scale, and was also hindered at the pilot scale. In the latter, we implicate the lower purity of N_2_ gas applied with a delay to achieve a quick drop of O_2_ (due to technical limitations) for not tackling PLases producers as efficiently as in the laboratory scale [26,28]. The production of phospholipases is a common feature of both Gram (+) or (−) bacteria. Secreted phospholipases are not only involved in phosphate and carbon metabolism but are also virulence factors for pathogenic species [35]. From the MYP (Mannitol Egg Yolk Polymyxin B) agar plates, we observed that isolates, which were unable to ferment mannitol but produced phospholipases, were “disappearing” from the agar plates over time. This observation enabled us to suggest that vegetative forms of *B. cereus*-types present in raw milk are one primary target of N_2_ treatment [26]. Moreover, observations from Mac Conkey agar plates, which support the growth of Gram (−) bacteria suggest that, over time, lactose fermenters were favoured to the detriment of non-fermenters during cold storage and N_2_ treatment, thereby indicating that significant changes occur at the population level in flushed milk [30]. As bacteria such as pseudomonads enter into the non-lactose-fermenter category, we assume that the effects of the N_2_ gas treatments are not Gram type-dependent.

Further studies are required to examine how some bacterial species circumvent the continuous N_2_ gas flushing as well as to identify the molecular mechanisms by which N_2_ exerts antibacterial effects against other bacterial species.

## 3. Experimental Section

### 3.1. Procurement of Raw and Pasteurised Milk Samples

The considered samples are listed in Table 2. For P3 and P6, 100 mL of milk were aseptically removed from 0.5 L milk containers, that were purchased from a local supermarket in Helsinki. Similarly, 100 mL of raw milks (L2R and L3R) and the corresponding pasteurised milks, L2P and L3P, were transferred into sterile bottles to be flushed shortly thereafter.

### 3.2. N_2_ Gas Treatments

The experimental system has been described [27]. Briefly, N_2_ gas (from AGA Ltd, Riimimäki, Finland) of 99.999% purity is introduced with a constant flow rate of 120 mL/min, into the headspace of flasks via 0.2-μm sterile filters (Schleicher & Schuell GmbH, Dassel, Germany). The outlet tube, which also holds a sterile filter, enables gas exchange with the environment. In this study, to enable the simultaneous N_2_ gas treatment of several samples, different flasks were connected in series via tubes holding 0.2-μm sterile filters. For the experiments with L2 and L3, N_2_ gas was first flushed through the raw milks (L2R and L3R) and then through the pasteurised milk samples (L2P and L3P). All milk flasks were placed on a multi-place magnetic stirrer (Variomag, Oberschleißheim, Germany) that stirred the milk at 220 rpm at regular intervals. All experiments were carried out at a storage temperature of 6 °C, with the exception of sample P6, which was stored at 8 °C. Constant temperature was maintained by partial immersion of the bottles in a refrigerated water bath (MGW Lauda MS/2) using ethanol (99.5%) as a cooling agent, with an immersion thermostat to ensure constant temperature. The pH of the milks was measured with a Metrohm 744 pH meter (Metrohm Ltd., Herisan, Switzerland) at the beginning and end of the experiments.

### 3.3. Microbiological Analyses of Milk

Initial bacterial counts were determined on the day of retail pasteurised milk purchase. When raw and pasteurised milks were simultaneously considered, raw milks underwent overnight cold storage at 3.5 °C prior to their analyses. The N_2_ gas flow was briefly interrupted during sample collection. A sample of 500 μL from control or flushed milks was drawn, serially diluted in saline solution (0.85% NaCl), and cultured on PCA (Plate count agar) (LabM, Ltd., Heywood, UK). To determine the “total plate counts”, 50 μL from serial dilutions were spread over agar plates. The plates were incubated at 30 °C for 3 days in aerobic conditions. Microbial analyses were performed for controls (C) or treated milks (N) at different intervals (usually after every 3rd day), and total plate counts were determined from three replicates. The production of extracellular enzymes was investigated by agar-diffusion assays [36,37]: clear transparent areas and dark blue areas around colonies were considered as positive reactions for protease and lipase activity respectively; the observation of an opaque zone around colonies was indicative of phospholipase production.

### 3.4. Evaluation of Aerobic Spore Levels at the End of the N_2_-Flushing

Two milliliters of the control and flushed milks from samples P3 and P6 were placed into test tubes and heat treated at 75 °C for 15 min in a water bath. Fifty microliters of the heat-treated fractions P3/C–P3/N and P6/C-P6/N were spread over BHI (Brain Heart Infusion, Oxoid Ltd., Cambridge, UK) plates and incubated at 30 °C for 3 days in aerobic conditions. The colonies were enumerated from three replicates.

### 3.5. DNA-Based Characterisation of Bacterial Isolates

#### 3.5.1. Selection of Isolates

For each milk sample, bacterial colonies present on PCA plates from the different sampling days were visually examined. Colonies with distinct aspects were purified, preserved on PCA agar slants at 5 °C, and also kept at −20 °C in BHI broth with 15% glycerol. Altogether 24 isolates (eight from each of the samples P3, P6 and L2P) were retained for further investigations (Table 1).

#### 3.5.2. DNA Extraction

Bacterial DNA was extracted from overnight cultures in BHI broth using the Wizard^®^ Genomic DNA Purification kit (Promega, Madison, WI, USA) according to the manufacturer’s guidelines.

#### 3.5.3. Partial Sequencing of the 16S rRNA and *rpoB* Genes

Amplifications of approximately 0.7 kb and 0.4 kb PCR fragments of the 16S rRNA and *rpoB* genes were performed with DyNAzyme II polymerase (Finnzymes, Espoo, Finland) and DNA Polymerase kit (Thermo Fischer, Finland) with the primers W01 and W012 [38], *rpoB*1698f and *rpoB*2041r [39], respectively. The sequencing reactions were performed with the primers W01 and *rpoB*1698f at the DNA Sequencing and Genomics Laboratory (Institute of Biotechnology/University of Helsinki). Sequences analyses were performed with BLAST (www.ncbi.nlm.nih.gov, [40]), and alignments were performed with Clustal-W (Mega 5, [41]).

## 4. Conclusions

The continuous flushing of N_2_ gas through the head space of bottles that contained pasteurised milks stored at 6 °C or 8 °C, showed high inhibitory effects on total bacterial counts in all experiments. When comparing retail pasteurised milks and milks pasteurised at the pilot plant scale, the treatments were most efficient when N_2_ was applied shortly after the pasteurisation stage. Moreover, the efficiency of the treatments at the level of pasteurised milk seemed to depend on the initial raw milk quality.

The analyses of the partial sequences of the 16S rRNA and *rpoB* genes revealed that the *Bacillus* species is common in milk under N_2_ treatment after prolonged cold storage. Irrespective of the conditions, whether C or N, *Bacillus weihenstephanensis* was present in all samples.

Indeed, the results obtained here indicate that N_2_ gas-based treatment of pasteurised milk constitutes an interesting approach to improve the quality of pasteurised milk and to extend the shelf life of pasteurised milk, although present data suggest that the treatments are temperature- and milk-age- (*i.e.*, “quality of raw milk”) -dependent. To the best of our knowledge, no study thus far has considered the use of N_2_ gas to treat pasteurised milks.

## Figures and Tables

**Table 1:** Spoilage features, 16S rRNA and *rpoB* gene nucleotide sequence-based characterization, of isolates from various pasteurized milk samples.

Isolates 1	Spoilage Features 2			Analyses of Gene Sequences					

				*16S rRNA*			*rpoB*		
	PR	LP	PL	Species	Acc. number in GenBank	% ID 3	Species	Acc. number in GenBank	% ID 3
**P3Nd3**	−	−	−	*Microbacterium lacticum*	NR_026160.1	99	ND		
				*Microbacterium schleiferi*	NR_044936.1	98			
				*Microbacterium flavum*	NR_041562.1				

**P3Na7**	+	−	+	*Bacillus weihenstephanensis*	NR_024697.1	99	*Bacillus weihenstephanensis*	CP000903.1	98
				*B. mycoides*	NR_036880.1				
				*B. thuringiensis*	NR_043403.1				
				*B. anthracis*	NR_041248.1				

**P3Ca14**	+	−	+	*B. weihenstephanensis*	NR_024697.1	99	*B. weihenstephanensis*	CP000903.1	98
				*B. mycoides*	NR_036880.1				
				*B. thuringiensis*	NR_043403.1				
				*B. anthracis*	NR_041248.1				

**P3Cb14**	+	−	+	*B. weihenstephanensis*	NR_024697.1	99	*B. weihenstephanensis*	CP000903.1	99
				*B. mycoides*	NR_036880.1	99			
				*B. thuringiensis*	NR_043403.1	98			
				*B. anthracis*	NR_041248.1	99			

**P3Na14**	+	−	+	*B. weihenstephanensis*	NR_024697.1	99	*B. weihenstephanensis*	CP000903.1	98
				*B. mycoides*	NR_036880.1				
				*B. thuringiensis*	NR_043403.1				
				*B. anthracis*	NR_041248.1				

**P3Nb14**	+	−	+	*B. weihenstephanensis*	NR_024697.1	99	*B.weihenstephanensis*	CP000903.1	99
				*B. mycoides*	NR_036880.1				
				*B. thuringiensis*	NR_043403.1				
				*B. anthracis*	NR_041248.1				

**P3Nc14**	+	−	+	*B. weihenstephanensis*	NR_024697.1	99	*B. weihenstephanensis*	CP000903.1	98
				*B. mycoides*	NR_036880.1				
				*B. thuringiensis*	NR_043403.1				
				*B. anthracis*	NR_041248.1				

**P3Nd14**	+	−	+	*B. weihenstephanensis*	NR_024697.1	99	*B. weihenstephanensis*	CP000903.1	99
				*B. mycoides*	NR_036880.1				
				*B. thuringiensis*	NR_043403.1				
				*B. anthracis*	NR_041248.1				

**P6Na14**	+	+	+	*B. weihenstephanensis*	NR_024697.1	99	*B. weihenstephanensis*	CP000903.1	98
				*B. mycoides*	NR_036880.1				
				*B. thuringiensis*	NR_043403.1				
				*B. anthracis*	NR_041248.1				

**P6Nb14**	+	+	+	*B. weihenstephanensis*	NR_024697.1	99	*B. weihenstephanensis*	CP000903.1	99
				*B. mycoides*	NR_036880.1				
				*B. thuringiensis*	NR_043403.1				
				*B. anthracis*	NR_041248.1				

**P6Nc14**	+	+	+	*B. weihenstephanensis*	NR_024697.1	99	*B. weihenstephanensis*	CP000903.1	99
				*B. mycoides*	NR_036880.1				
				*B. thuringiensis*	NR_043403.1				
				*B. anthracis*	NR_041248.1				

**P6Nd14**	+	+	+	*B. weihenstephanensis*	NR_024697.1	99	*B. weihenstephanensis*	CP000903.1	98
				*B. mycoides*	NR_036880.1				
				*B. thuringiensis*	NR_043403.1				
				*B. anthracis*	NR_041248.1				

**P6Ne14**	+	+	+	*B. weihenstephanensis*	NR_024697.1	99	*B. weihenstephanensis*	CP000903.1	99
				*B. mycoides*	NR_036880.1				
				*B. thuringiensis*	NR_043403.1				
				*B. anthracis*	NR_041248.1				

**P6Nf14**	+	+	+	*B. weihenstephanensis*	NR_024697.1	99	ND		
				*B. mycoides*	NR_036880.1				
				*B. thuringiensis*	NR_043403.1				
				*B. anthracis*	NR_041248.1				

**P6Ng14**	+	+	+	*B. weihenstephanensis*	NR_024697.1	99	*B. weihenstephanensis*	CP000903.1	99
				*B. mycoides*	NR_036880.1				
				*B. thuringiensis*	NR_043403.1				
				*B. anthracis*	NR_041248.1				

**P6Nh14**	+	+	+	*B. weihenstephanensis*	NR_024697.1	100	ND		
				*B. mycoides*	NR_036880.1	99			
				*B. thuringiensis*	NR_043403.1				
				*B. anthracis*	NR_041248.1				

**L2PNa**	+	+	−	*B. sonorensis*	NR_025130.1	98	ND		
				*B. aerius*	NR_042338.1				

**L2PNb**	+	+	+	*B. weihenstephanensis*	NR_024697.1	99	*B. cereus*	EF607283.1	99
				*B. mycoides*	NR_036880.1		*B. thuringiensis*	CP001903.1	100
				*B. thuringiensis*	NR_043403.1		*B. cereus*	FJ188319.1	99
				*B. anthracis*	NR_041248.1		*B. cereus*	AE016877.1	100

**L2PNa6**	+	+	−	*B. sonorensis*	NR_025130.1	98	*B. licheniformis*	CP000002.3	96
				*B. subtilis ssp.* subtilis	NR_027552.1		*B. licheniformis*	AE017333.1	96

**L2PNa9**	+	+	−	*B. aerophilus*	NR_042339.1	99	ND		
				*B. pumilus*	NR_043242.1				

**L2PNa13**	ɛ	+	−	*B. sonorensis*	NR_025130.1	98	ND		
				*B. subtilis ssp.* subtilis	NR_027552.1	97			

**L2PNa17**	+	+	+	*B. weihenstephanensis*	NR_024697.1	99	*B. weihenstephanensis*	CP000903.1	99
				*B. mycoides*	NR_036880.1				
				*B. thuringiensis*	NR_043403.1				
				*B. anthracis*	NR_041248.1				

**L2PCa21**	−	+	−	*Paenibacillus odorifer*	NR_028887.1	99	*Paenibacillus odorifer*	AY493862.1	99

**L2PNa27**	+	+	+	*B. weihenstephanensis*	NR_0246971	99	*B. weihenstephanensis*	CP000903.1	99
				*B. mycoides*	NR_036880.1				
				*B. thuringiensis*	NR_043403.1				
				*B. anthracis*	NR_041248.1				

1 P3, P6 and L2P correspond to the investigated pasteurised milk samples; C = control, N = N_2_ flushing; a, b = order of selection of the isolates; 0 to 27 = days of cold storage;

2 + = positive, − = negative, ɛ = weak enzyme production;

3 only values equal or above 98% (with exception of L2PNa6).

**Table 2:** Pasteurised and raw milks.

Milk samples	Origin
P3	Retail pasteurised milk (Arla Ingman Ltd, Finland)
P6	Retail pasteurised milk (Arla Ingman Ltd, Finland)
L2R	Raw milk
L2P	Raw milk L2R pasteurised (73 °C/15 s) at the pilot plant / Univ. of Helsinki
L3R	Raw milk
L3P	Raw milk L3R pasteurised (73 °C/15 s) at the pilot plant / Univ. of Helsinki

## References

[1] Newell D.G., Koopmans M., Verhoef L., Duizer E., Aidara-Kane A., Sporng H., Opsteegh M., Langelaar M., Threfall J., Scheutz F. (2010). Food-borne diseases. The challenges of 20 years ago still persist while new ones continue to emerge. Int. J. Food Microbiol.

[2] Godfray H.C., Beddington J.R., Crute I.R., Haddad L., Lawrence D., Muir J.F., Pretty J., Robinson S., Thomas S.M., Toulmin C. (2010). Food security: the challenge of feeding 9 billion people. Science.

[3] Tudge C. (2012). Enlightened agriculture. Food Ethics Council.

[4] Gustavsson J., Cederberg C., Sonesson U., van Otterdijk R., Meybeck A Global Food Losses and Food Waste.

[5] DEFRA (2012). http://www.defra.gov.uk.

[6] Chambers J.V., Robinson R.K. (2002). The Microbiologyof Raw Milk. Dairy Microbiology Handbook.

[7] Munsch-Alatossava P., Alatossava T. (2007). Antibiotic resistance of raw-milk associated psychrotrophic bacteria. Microbiol. Res.

[8] Munsch-Alatossava P., Gauchi J.P., Chamlagain B., Alatossava T (2012). Trends of antibiotic resistance in mesophilic and psychrotrophic bacterial populations during cold storage of raw milk. ISRN Microbiol..

[9] Munsch-Alatossava P., Ikonen V., Alatossava T., Gauchi J.P., Pana M. (2012). Trends of Antibiotic Resistance (AR) in Mesophilic and Psychrotrophic Bacterial Populations During Cold Storage of Raw Milk, Produced by Organic and Conventional Farming Systems. Antibiotic Resistant Bacteria, A Continuous Challenge in the New Millenium.

[10] Meunier-Goddik L., Sandra S, Roginski H., Fuquay J.W., Fox P.F. (2002). Pasteurised Milk Products. Encyclopedia of Dairy Sciences.

[11] Olivier S.P., Jayarao B.M., Almeida R.A. (2005). Foodborne pathogens in milk and the dairy farm environment: Food safety and public health implications. Foodborne Pathog. Dis.

[12] U.S. Food and Drug Administration (2005). Grade “A” Pasteurised milk ordinance (PMO).

[13] Hillerton J.E., Berry E.A. (2004). Quality of the milk supply: European regulations versus practice. NMC Annual meeting Proceedings.

[14] Ranieri M.L., Huck J.R., Sonnen M., Barbano D.M., Boor K.J. (2012). High temperature, short time pasteurization temperatures inversely affect bacterial numbers during refrigerated storage of pasteurised fluid milk. J. Dairy Sci.

[15] Petrus R.R., Loiola C.G., Oliveira C.A.F. (2009). Microbiological shelf life of pasteurised milk in bottle and pouch. J. Food Sci.

[16] Ranieri M.L., Boor K.J. (2009). Short communication: Bacterial ecology of high temperature, short time pasteurized milk processed in the United States. J. Dairy Sci.

[17] Ivy R.A., Ranieri M.L., Martin N.H., Den Bakker H.C., Xavier B.M., Wiedmann M., Boor K.J. (2012). Identification and characterization of psychrotolerant sporeformers associated with fluid milk production and processing. Appl. Environ. Microbiol.

[18] Huck J.R., Sonne M., Boor K.J. (2009). Tracking heat-resistant, cold thriving fluid milk spoilage bacteria from farm to packaged product. J. Dairy Sci.

[19] Silliker J.H., Wolfe S.K. (1980). Microbiological safety considerations in controlled-atmosphere storage of meats. Food Technol.

[20] King J.S., Mabbitt L.A. (1982). Preservation of raw milk by the addition of carbon dioxide. J. Dairy Res.

[21] Hotchkiss J.H., Lee E. (1996). Extending the shelf life of dairy products with dissolved carbon dioxide. Eur. Dairy Mag.

[22] Ruas-Madieodo P., Bada-Gancedo J.C., Fernandez-Garcia E., Gonzalez de Lano D., de Los Reyes-Gavilan C.G. (1996). Preservation of the microbiological and biochemical quality of raw milk by carbon dioxide addition: A pilot- scale study. J. Food Prot.

[23] Rajagopal M., Werner B.G., Hotchkiss J.H. (2005). Low pressure CO_2_ storage of raw milk: Microbiological effects. J. Dairy Sci.

[24] Dechemi S., Benjelloun H., Lebeault J.M. (2005). Effect of modified atmospheres on the growth and extracellular enzymes of psychrotrophs in raw milk. Eng. Life Sci.

[25] Murray S.K., Kwan K.K.H., Skura B.J., Mc Kellar R.C. (1983). Effect of nitrogen flushing on the production of proteinase by psychrotrophic bacteria in raw milk. J. Food Sci.

[26] Munsch-Alatossava P., Gursoy O., Alatossava T. (2010). Exclusion of phospholipases (PLs)-producing bacteria in raw milk flushed with nitrogen gas (N_2_). Microbiol. Res.

[27] Munsch-Alatossava P., Gursoy O., Alatossava T. (2010). Potential of nitrogen gas (N_2_) to control psychrotrophs and mesophiles in raw milk. Microbiol. Res.

[28] Munsch-Alatossava P., Gursoy O., Alatossava T (2011). Improved storage of cold raw milk by continuous flushing of N_2_ gas separated from compressed air: A pilot scale study. J. Food Process. Technol..

[29] Munsch-Alatossava P., Alatossava T, Cohen G.E., Levin C.M. (2011). Controlled Atmosphere-based Improved Storage of Cold Raw Milk: Potential of N_2_ Gas. Food Storage.

[30] Munsch-Alatossava P., Ghafar A., Gursoy O., Alatossava T Changes in bacterial populations present in raw and pasteurised milks under N_2_ gas flushing.

[31] Berge O., Guinebretière M.H., Achouak W., Normand P., Heulin T. (2002). *Paenibacillus graminis* sp. nov. and *Paenibacillus odorifer* sp. nov, isolated from plant roots, soil and food. Int. J Syst. Evol. Microbiol.

[32] Da Mota F.F., Gomes E.A., Paiva E., Rosado A.S., Seldin L. (2004). Use of *rpoB* gene analysis for identification of nitrogen-fixing *Paenibacillus* species as an alternative to the 16S rRNA gene. Lett. Appl. Microbiol.

[33] Lechner S., Mayr R., Francis K.P., Prüβ B.M., Kaplan T., Wieβner-Gunkel E., Stewart G.S.A.B., Scherer S. (1998). *Bacillus weihenstephanensis* sp. nov. is a new psychrotolerant species of the *Bacillus cereus* group. Int. J. Syst. Bacteriol.

[34] Réjasse A., Gilois N., Barbosa I., Huillet E., Bevilacqua C., Tran S., Ramarao N., Stenfors Arnesen L.P., Sanchis V. (2012). Temperature-dependent production of various PlcR-controlled virulence factors in *Bacillus weihenstephanensis* strain KBAB4. Appl. Environ. Microbiol.

[35] Schmiel D.H., Miller V.L. (1999). Bacterial phospholipases and pathogenesis. Microbes Infect.

[36] Dogan B., Boor K.J. (2003). Genetic diversity and spoilage potentials among *Pseudomonas* spp. isolated from fluid milk products and dairy processing plants. Appl. Environ. Microbiol.

[37] Munsch-Alatossava P., Alatossava T. (2006). Phenotypic characterization of raw-milk associated psychrotrophic bacteria. Microbiol. Res.

[38] Ogier J.C., Son O., Gruss A., Tailliez P., Delacroix-Buchet A. (2002). Identification of the bacterial microflora in dairy products by temporal temperature gradient gel electrophoresis. Appl. Environ. Microbiol.

[39] Dahllöf I., Baillie H., Kjelleberg S. (2000). *rpoB*-based microbial community analysis avoids limitations inherent in 16S rRNA gene intraspecies heterogeneity. Appl. Environ. Microbiol.

[40] Altschul S.F., Gish W., Miller W., Myers E.W., Lipman D.J. (1990). Basic local alignment search tool. J. Mol. Biol.

[41] Tamura K., Peterson D., Peterson N., Stecher G., Nei M., Kumar S. (2011). Mega 5: Molecular evolutionary genetics analysis using maximum likelihood, evolutionary distance, and maximum parsimony methods. Mol. Biol. Evol.

